# Internal tremor in people with Parkinson’s Disease: Demographic characteristics and comorbid symptoms

**DOI:** 10.1016/j.prdoa.2023.100229

**Published:** 2023-11-07

**Authors:** Lana M. Chahine, Lakshmi Arbatti, Abhishek Hosamath, Amy Amara, Karen E. Anderson, Jennifer Purks, Shirley Eberly, Daniel Kinel, Sneha Mantri, Soania Mathur, David Oakes, David G. Standaert, Daniel Weintraub, Ira Shoulson, Connie Marras

**Affiliations:** a Department of Neurology, University of Pittsburgh, Pittsburgh, PA, the United States of America; b Grey Matter Technologies, a wholly owned subsidiary of Modality.ai, San Francisco, CA, the United States of America; c Department of Neurology, University of Colorado Anschutz Medical Campus, Aurora, CO, the United States of America; d Departments of Psychiatry and Neurology, Georgetown University, Washington, DC, the United States of America; e Center for Health + Technology, University of Rochester Medical Center, Rochester, NY, the United States of America; f Department of Neurology, University of Rochester Medical Center, Rochester, NY, the United States of America; g Department of Biostatistics and Computational Biology, University of Rochester, Rochester, NY, the United States of America; h Department of Neurology, Duke University, Durham, NC, the United States of America; i PD Avengers, Toronto, Canada; j Department of Neurology, University of Alabama at Birmingham, Birmingham, AL, the United States of America; k Departments of Psychiatry and Neurology, Perelman School of Medicine at the University of Pennsylvania, Philadelphia, the United States of America; l Edmond J Safra Program in Parkinson’s Disease, University Health Network, University of Toronto, Toronto, Canada

## Abstract

**Introduction:**

Internal tremor (IT) occurs in > 30 % of people with Parkinson’s Disease (PwPD), but remains largely uninvestigated. Our objective was to describe demographic characteristics and associated symptoms in PwPD who reported IT.

**Methods:**

This was a matched case-control survey study. Data were from PwPD in the Fox Insight study who answered the Patient Report of Problems (PD-PROP) assessment, a series of open-ended questions that asks people to report in their own words their most bothersome PD-related problems. Cases were those who reported IT ≥ 1 times compared with PwPD controls who did not report IT and were matched 1:3 by age and disease duration.

**Results:**

243 PwPD reported IT as a bothersome problem. Mean (SD) age of cases was 64.9 (9.4) years and disease duration was 3.8 (4.0) years. The proportion of women was greater among cases compared to controls (74 % vs 47 %, p < 0.0001). External tremor as a PD-PROP symptom was reported by 98 % cases and 48 % controls (p < 0.0001). Several non-motor symptoms were more common among cases than controls, including anxiety (35 % vs 20 %), fatigue (41 % vs 31 %), and pain (57 % vs 37 %). The odds of IT was significantly higher in women when adjusting for anxiety and motor experiences of daily living score (OR 3.07, 95 %CI 2.14–4.41, p < 0.0001).

**Conclusion:**

PwPD with IT report a range of associated symptoms, including external tremor, anxiety, and pain. Sex differences in the experience of IT may exist. Studies of IT are needed to understand its etiology and inform clinical care.

## Highlights

• Internal tremor is among the most bothersome symptoms for some PD patients.
• PD patients with internal tremor are more likely to report bothersome external tremor.
• Patients with internal tremor are more likely to report a range of nonmotor symptoms.
• In one-third of patients with internal tremor, anxiety is also a bothersome symptom.
• Sex differences in the experience of IT may exist.

## 1 Introduction

Internal tremor (IT) is the report of a feeling of inner tremor by an individual even though there may not be commensurate external, visible features. While it is not specific to Parkinson’s Disease (PD), it may be common in people with PD (PwPD), occurring in 32–44 %[1], [2], and in some individuals it is the presenting symptom of the disease[2]. Phrases such as “shaking inside”[3], “vibration”[3], and even an “internal earthquake”[4] are used by PwPD to describe IT.

There are few data on the experience of IT in PD. While IT may be a motor phenomenon, it could also be a non-motor symptom, related to anxiety, sensory, or autonomic symptoms. Many commonly administered patient- and clinician-reported outcome scales do not screen for IT. Assessments that allow PwPD to report their symptoms in free-text form offer the opportunity to explore PD manifestations beyond what is assessed in categorical scales. Indeed, in a study that asked PwPD to report their most bothersome symptoms[3], we found that IT was reported by some individuals as their most bothersome problem. Given the gaps in knowledge surrounding IT in PD, our objectives were to describe demographic characteristics and associated symptoms in PwPD reporting IT as one of their most bothersome symptoms.

## 2 Methods

This was a matched case-control survey study. Data were from Fox Insight (FI)[5], an online longitudinal observational study that collects patient-reported outcomes from > 25,000 individuals self-identified as having PD. This study is approved by the WCG IRB, and informed consent is obtained from each participant. Data used in the preparation of this article were obtained from the Fox Insight database on February 3, 2020. For up-to-date information on the study, visit https://foxinsight-info.michaeljfox.org.

## 3 Sample

The sample was comprised of PwPD participating in FI who had completed a Patient Report of Problems (PD-PROP) assessment. As described[3], PD-PROP is a series of open-ended questions including “What is the most bothersome problem for you due to your Parkinson’s disease?”. Respondents report their five most bothersome problems and their impact on function. Inclusion criteria were (1) ≥ 1 response to PD-PROP (2) available data on age, sex and disease duration.

Cases were defined as individuals who reported IT at ≥ 1 PD-PROP assessment. Controls were defined as individuals who never reported IT. For each case, three controls were matched by age, disease duration (years) and the PD-PROP visit number at which the case first reported IT. Data from the first FI visit for each participant who met the aforementioned criteria were used for this analysis.

## 4 Assessments

Assessments administered in FI that were included in this analysis are as follows:

-Demographics: age, biological sex, race/ethnicity, education

-Age at PD onset and year of diagnosis.

-PD-PROP: PD-PROP responses are analyzed via an algorithm that converts verbatim (free-text) responses into classified symptoms using a combination of human curation, natural language processing and machine learning[3]. Each verbatim response is assigned a symptom category that is a component of a specific motor or non-motor domain. The domain of Tremor includes two individual symptoms: tremor (excluding IT; hereto forth referred to as external tremor), and IT. For the purpose of curation and symptom assignment, IT was defined as invisible rhythmic sensation and excludes jitteriness, restlessness, or skin-crawling sensation.

-Geriatric Depression Scale-15-item[6]. A score ≥ 5 was considered to indicate depression.

–Non-Motor Symptoms Questionnaire (NMS-Quest)[7].

-Movement Disorders Society Unified Parkinson’s Disease Rating Scale part II (MDS-UPRDS II)[8].

NMS-Quest and MDS-UPDRSII assessments available within 30 days before or after PD-PROP visit were included in this analysis.

-PD medications: participants select prescribed PD medications from a list presented to them at each FI study visit.

### 4.1 Statistical analysis

Sample characteristics were summarized with basic descriptive statistics (mean (SD) for continuous variables; frequencies and percentages for categorical variables). Proportions between groups were compared using chi-squared test, Fisher’s exact test, or Cochran-Armitage trend test, as appropriate. Proportions of cases and controls reporting symptoms within each of the 9 domains of the PD-PROP were first compared. When the proportion reporting symptoms within a given domain was found to be significantly different between groups, the proportion reporting each symptom within that domain was then compared. For the tremor domain, only the tremor symptom was compared between the case and control groups. Proportions endorsing each symptom on the NMS-Quest was also compared between groups. Post hoc, cases and controls were compared adjusting for sex and MDS-UPDRS II total score. For analyses comparing symptoms between cases and controls, false discovery rate (FDR) p-value correction based on Benjamini and Hochberg procedure was applied to adjust for multiple comparisons.

Cases were then stratified by the presence or absence of anxiety as reported on the PD-PROP, and separately by sex, and the above analyses were repeated. Post-hoc, to examine the relationship of sex and IT adjusting for anxiety, a conditional logistic regression was conducted with IT as the outcome and sex, anxiety, and MDS-UPDRSII as covariates.

All analyses were conducted with SAS version 9.4 except FDR p-value correction which was conducted at https://www.sdmproject.com.

## 5 Results

The sample included 243 cases reporting IT at any PD-PROP visit, and 729 matched controls. Sample characteristics are shown in Table 1. Among cases, mean (SD) age was 64.9 (9.4) years and disease duration was 3.8 (4.0) years. The proportion of women was significantly greater among cases compared to controls (74 % vs 47 %, p < 0.0001). A similar proportion of cases and controls reported being prescribed each of four PD medication categories (Table 1).

**Table 1:** Subject characteristics at baseline.

	INTERNAL TREMOR	CONTROLS	P-VALUE
	(N = 243)	(N = 729)	
Age:			–
<50	16 (7 %)	48 (7 %)	
50 – 59	54 (22 %)	162 (22 %)	
60 – 69	76 (31 %)	228 (31 %)	
70 – 79	88 (36 %)	264 (36 %)	
>= 80	9 (4 %)	27 (4 %)	
Years Since Diagnosis:			–
< 3	116 (48 %)	348 (48 %)	
3 – 5	67 (28 %)	201 (28 %)	
6 – 7	29 (12 %)	87 (12 %)	
8 – 9	13 (5 %)	39 (5 %)	
>= 10	18 (7 %)	54 (7 %)	
Sex:			<0.0001
Women	181 (74 %)	341 (47 %)	
Men	62 (26 %)	388 (53 %)	
Education:			0.45
High School	25 (11 %)	62 (9 %)	
Associates/College	132 (56 %)	401 (56 %)	
Post Graduate	79 (33 %)	250 (35 %)	
MDS-UPDRS II (Quartiles):			0.07
Q1: 0 – 6	87 (40 %)	210 (32 %)	
Q2: 7 – 11	60 (27 %)	194 (30 %)	
Q3: 12 – 17	42 (19 %)	152 (23 %)	
Q4: >=18	30 (14 %)	99 (15 %)	
GDS-15 score > 5			0.51
No	103 (64 %)	324 (66 %)	
Yes	59 (36 %)	164 (34 %)	
PD Medications*			
Levodopa	166 (75 %)	496 (74 %)	0.77
Dopamine Agonist	57 (26 %)	179 (27 %)	0.78
MAO-B Inhibitor	48 (22 %)	167 (25 %)	0.33
Other (carbidopa, Entacapone, or tolcapone)	10 (5 %)	23 (3 %)	0.46

*Self-reported medication data were available for 220 cases and 666 controls.

## 6 Comparison of associated symptoms in cases and controls

Among cases, 237 (98 %) reported external tremor as one of their other most bothersome symptoms on the PD-PROP, compared to 349 (48 %) controls (FDR-adjusted p = 0.0002). Table 2 shows other most bothersome domains reported on the PD-PROP. A greater proportion of cases reported anxiety compared to controls (35 % vs 20 % respectively, FDR-adjusted p < 0.004). Other symptoms within each of the domains found to be significantly different are shown in supplementary Table S1. In unadjusted analysis, cases were more likely to report the following symptoms as being bothersome to them compared to controls (% cases vs controls respectively): negative emotions or thoughts (28 %, 20 %), physical fatigue (41 %, 31 %), pain (57 %, 37 %), and cramp/spasm (19 %, 12 %). However, after adjusting for sex and MDS-UPDRS II score, only tremor, anxiety/worry, and pain remained significantly different between the groups (Supplementary Table S1).

**Table 2:** Domains of other most bothersome symptoms reported on the PD-PROP by cases and controls. Values shown are N (%) reported.

	INNER TREMOR (N = 243)	CONTROLS (N = 729)	P-VALUE	ADJUSTED P-VALUE*
Anxiety/Worry	86 (35 %)	144 (20 %)	<0.001	0.001
Affect/Motivation	149 (61 %)	292 (40 %)	<0.001	<0.001
Autonomic Dysfunction	82 (34 %)	189 (26 %)	0.043	0.14
Bradykinesia	54 (22 %)	181 (25 %)	0.57	0.50
Cognition	79 (33 %)	207 (28 %)	0.34	0.46
Dyskinesias	12 (5 %)	31 (4 %)	0.70	0.81
Fatigue	101 (42 %)	224 (31 %)	0.005	0.020
Fluctuations	20 (8 %)	38 (5 %)	0.16	0.46
Gait	84 (35 %)	267 (37 %)	0.65	0.46
Other Motor	123 (51 %)	377 (52 %)	0.77	0.81
Pain	141 (58 %)	270 (37 %)	<0.001	<0.001
Postural Instability	61 (25 %)	227 (31 %)	0.14	0.06
Rigidity	55 (23 %)	152 (21 %)	0.65	0.94
Sleep	96 (40 %)	197 (27 %)	<0.001	0.020

*Adjusted for sex and MDS-UPDRS II score.

NMS-Quest data were available for 217 cases and 650 controls (Supplementary Table S2). Similar to findings from the PD-PROP, symptoms more often reported on the NMS-Quest by cases compared to controls in unadjusted analyses were (% cases vs controls respectively): vomiting/nausea (36 %, 22 %), unexplained pain (49 %, 38 %), sweating (32 %, 22 %), and hallucinations (15 %, 8 %). Only hallucinations remained significantly different in the two groups when adjusting for sex and MDS-UPDRS II score.

We next stratified cases based on the presence (n = 157) and absence (n = 86) of anxiety as reported on the PD-PROP. Cases with anxiety were more likely to report depression symptoms on the PD-PROP (14 % vs 4 %, p = 0.008). Otherwise, demographic characteristics and other most bothersome symptoms did not differ significantly between the groups (Supplementary Table S3). Stratifying cases by sex, women with IT were older compared with men, but otherwise there were no significant differences (Supplementary Table S4).

In the sample as a whole, the odds of reporting IT were higher in women compared with men when adjusting for anxiety and MDS-UPDRSII (Odds ratio (OR) = 3.07, 95 % confidence interval (CI) 2.14–4.41, p < 0.0001), but anxiety remained significantly associated with odds of IT in this model (OR = 1.71, 95 %CI 1.21–2.42, p = 0.003). MDS-UPDRSII was not independently associated with odds of IT (OR = 0.99, 95 %CI 0.97–1.02, p = 0.50).

## 7 Discussion

Internal tremor is experienced by some PwPD as among their most bothersome symptoms[3]. We found that PwPD who report IT as a bothersome symptom are more likely to be women and to have a profile of comorbid motor and non-motor symptoms that differs from those who do not report IT.

There are few data on the prevalence of IT in PD. In a study by Shulman et al[2] that screened 100 PwPD and 50 controls for symptoms of IT, 44 % of PwPD and 6 % of controls endorsed IT of any severity. In some, it was the presenting feature of the disease, and in the majority occurred within 1 year of disease onset. In another study that screened 89 PwPD for IT, 33 % reported the symptom[1]. In the studied sample, the prevalence based on spontaneous report as one of a person’s five most bothersome symptoms was 0.5 %[3]. Although this is not a high proportion, it does demonstrate that it can be an important source of distress.

The etiology of IT remains largely unknown. Our study does not allow us to establish causality, but the pattern of comorbid symptoms reported is informative. IT could be a motor symptom, a tremor that is too low in amplitude to be externally observed. This hypothesis is supported by the fact that almost all individuals reporting IT also reported external tremor, in contrast with only half of those not reporting IT. In a study[1] investigating IT in people with PD, multiple sclerosis, and essential tremor, those who experienced external tremor were also more likely to report IT, but IT was also endorsed in 29 % of individuals without external tremor. In future studies, it would be informative to compare activation conditions (rest vs action) and history of comorbid ET diagnosis in PwPD with versus without IT. In addition, electrophysiologic studies are needed to help clarify whether IT is a form fruste of external motor tremor in some individuals.

On the other hand, it is possible that IT is a non-motor manifestation. In our sample, cases were more likely to report anxiety as among their most bothersome symptoms. Similarly, Shulman et al[2] found that about two-thirds of individuals with IT reported an associated sense of anxiety. While IT could be a symptom of anxiety, it also remains possible that individuals with anxiety are more likely to perceive and report more symptoms[9], [10]. Consistent with this hypothesis, we found that cases were more likely to report a number of additional symptoms. In addition, the sense of IT could lead to anxiety. However, a substantial proportion of those reporting IT explicitly deny anxiety[1]. It is also possible that IT may be sensory symptom or a consequence of autonomic dysfunction. In our cohort, pain was significantly more common in cases than controls, and in the Shulman et al study[2], sensory symptoms such as tingling and aching were also more common in PwPD with versus without IT[2]. A range of autonomic symptoms were also more common in cases compared to controls in our sample, but these differences may at least in part be explained by effects of sex and disease severity.

Cases were more likely to be women compared to controls. Given that women with PD are more likely to experience anxiety and sensory symptoms[11], these results are not surprising, but we found that women were still significantly more likely to have bothersome IT after adjusting for anxiety. Our findings add to the growing body of literature on sex differences in the experience of PD[12].

Internal tremor is more common in the medication “off” state, and has been reported as a manifestation of non-motor fluctuations[2], [13]. In our sample, motor fluctuations and dyskinesias were not reported more often as bothersome symptoms in cases compared to controls. There were no differences in types of PD medications taken by cases and controls, but we do not have data on medication dosage and frequency.

Strengths of this study include the large sample size with both cases and controls having a range of disease durations. Limitations include the lack of data on disease severity aside from the MDS-UPDRSII. We analyzed data only from a single visit for each participant, and longitudinal analysis of those reporting IT would be informative. Lack of racial and ethnic diversity and the high level of education of the FI cohort limit generalizability. IT may be under-reported by PwPD and there may be controls who experience IT but just did not rank it as among their most bothersome symptoms. In addition, diagnosis in FI is self-reported, but data indicate this self-reported diagnosis of PD is usually accurate[14].

In conclusion, some PwPD report IT among their most bothersome PD symptoms and experience a range of other symptoms including not only external tremor but also anxiety and pain. Given the high prevalence of other symptoms in individuals with IT compared to controls, IT in PwPD may be a marker of greater symptom burden. Educating clinicians and patients about IT may improve its detection. Investigations of IT in PD are needed to better understand its etiology, natural history, and to inform clinical care.

## Declaration of Competing Interest

The authors declare that they have no known competing financial interests or personal relationships that could have appeared to influence the work reported in this paper.
