# Supplementary material for: Internal tremor in people with Parkinson’s Disease: Demographic characteristics and comorbid symptoms
Source: Clin Park Relat Disord. 2023 Nov 7;9:100229. doi: 10.1016/j.prdoa.2023.100229 (PMC10689282; doi:10.1016/j.prdoa.2023.100229)
Supplement: Supplementary data 1 [file mmc1.docx]

**Supplementary Table 1**: Other most bothersome symptoms reported on the PD-PROP by cases and controls, for the domains that were significantly different between the groups. Values shown are N (%) reported.

|  | INNER TREMOR  (N = 243) | CONTROLS  (N = 729) | P-VALUE | ADJUSTED P-VALUE* |
| --- | --- | --- | --- | --- |
| **Tremor** |  |  |  |  |
| Tremor | 237 (98%) | 349 (48%) | 0.001 | 0.002 |
| **Affect/Motivation** |  |  |  |  |
| Anxiety/Worry | 86 (35%) | 144 (20%) | 0.001 | 0.004 |
| Negative Emotions | 69 (28%) | 148 (20%) | 0.040 | 0.08 |
| Loneliness/Isolation | 5 (2%) | 16 (2%) | 1.00 | 0.99 |
| Depressive Symptoms | 19 (8%) | 61 (8%) | 0.97 | 0.99 |
| Apathy | 13 (5%) | 27 (4%) | 0.53 | 0.37 |
| Pseudobulbar Affect | 1 (<0.5 %) | 1 (<0.5 %) | 0.78 | 0.76 |
| Hallucinations | 2 (1%) | 4 (1%) | 0.91 | 0.59 |
| Impulse Control | 0 (0%) | 3 (<0.5%) | 0.89 | 0.99 |
| Delusions/Psychosis | 2 (1%) | 0 (0%) | 0.17 | 0.99 |
| Death/Suicidal Ideation | 0 (0%) | 1 (<0.5%) | 1.00 | 0.99 |
| **Autonomic Dysfunction** |  |  |  |  |
| Bladder Incontinence | 9 (4%) | 37 (5%) | 0.70 | 0.52 |
| Swallowing Problems | 14 (6%) | 25 (3%) | 0.27 | 0.29 |
| Excessive Sweating | 5 (2%) | 6 (1%) | 0.35 | 0.56 |
| Temperature Dysregulation | 6 (2%) | 3 (<0.5%) | 0.040 | 0.09 |
| Sexual Dysfunction | 2 (1%) | 10 (1%) | 0.97 | 0.99 |
| Altered Bowel Frequency | 33 (14%) | 94 (13%) | 0.97 | 0.99 |
| Lightheadedness/Dizziness | 12 (5%) | 23 (3%) | 0.44 | 0.93 |
| Bloating | 6 (2%) | 14 (2%) | 0.89 | 0.99 |
| Nausea | 5 (2%) | 8 (1%) | 0.64 | 0.72 |
| Frequent Urination | 5 (2%) | 20 (3%) | 0.89 | 0.99 |
| Diarrhea | 1 (<0.5%) | 3 (<0.5%) | 1.00 | 0.99 |
| Abdominal Discomfort | 6 (2%) | 1 (<0.5%) | 0.012 | 0.24 |
| Bowel Incontinence | 0 (0%) | 2 (<0.5%) | 1.00 | 0.99 |
| Bowel Urgency | 0 (0%) | 0 (0%) | -- | -- |
| **Fatigue** |  |  |  |  |
| Physical Fatigue | 100 (41%) | 223 (31%) | 0.019 | 0.08 |
| Mental Fatigue | 2 (1%) | 5 (1%) | 1.00 | 0.99 |
| **Pain** |  |  |  |  |
| Headache | 6 (2%) | 2 (<0.5%) | 0.022 | 0.078 |
| Pain/Discomfort | 138 (57%) | 269 (37%) | 0.001 | 0.002 |
| Cramp/Spasm | 46 (19%) | 85 (12%) | 0.022 | 0.09 |
| **Sleep** |  |  |  |  |
| Poor Sleep Quality | 49 (20%) | 105 (14%) | 0.10 | 0.17 |
| Sleep Maintenance Insomnia | 30 (12%) | 53 (7%) | 0.05 | 0.37 |
| Excessive Daytime Sleepiness | 15 (6%) | 37 (5%) | 0.86 | 0.99 |
| RBD-Like Symptoms | 3 (1%) | 11 (2%) | 1.00 | 0.99 |
| Sleep Onset Insomnia | 8 (3%) | 11 (2%) | 0.27 | 0.76 |
| Dreams | 4 (2%) | 9 (1%) | 0.97 | 0.99 |
| RLS Restlessness | 10 (4%) | 11 (2%) | 0.05 | 0.17 |
| Parasomnia Unspecified | 0 (0%) | 0 (0%) | -- | -- |
| Early Morning Awakening | 1 (<0.5%) | 4 (1%) | 1.00 | 0.99 |

*Adjusted for sex and MDS-UPDRS II score

**Supplementary table 2**: Symptoms endorsed on the NMS-QUEST in case and controls*. Values shown are N (%) reported.

|  | INNER TREMOR  (N = 217) | CONTROLS  (N = 650) | P-VALUE | ADJUSTED  P-VALUE** |
| --- | --- | --- | --- | --- |
| Sialorrhea | 47 (22%) | 172 (26%) | 0.34 | 0.792 |
| Taste changes | 69 (32%) | 202 (31%) | 0.84 | 0.81 |
| Swallowing difficulty | 72 (33%) | 181 (28%) | 0.3 | 0.43 |
| Vomiting or nausea | 79 (36%) | 138 (21%) | 0.003 | 0.09 |
| Constipation | 122 (56%) | 335 (52%) | 0.39 | 0.52 |
| Bowel Incontinence | 30 (14%) | 71 (11%) | 0.39 | 0.45 |
| Incomplete bowel Emptying | 101 (47%) | 293 (45%) | 0.79 | 0.81 |
| Urinary urgency | 142 (65%) | 435 (67%) | 0.79 | 0.79 |
| Nocturia | 147 (68%) | 462 (71%) | 0.50 | 0.68 |
| Unexplained Pains | 107 (49%) | 250 (38%) | 0.037 | 0.27 |
| Changes in weight | 33 (15%) | 67 (10%) | 0.15 | 0.21 |
| Memory changes | 101 (47%) | 296 (46%) | 0.84 | 0.56 |
| Apathy | 82 (38%) | 225 (35%) | 0.55 | 0.33 |
| Hallucinations | 33 (15%) | 52 (8%) | 0.030 | 0.009 |
| Difficulty concentrating | 112 (52%) | 297 (46%) | 0.30 | 0.18 |
| Depressed mood | 119 (55%) | 351 (54%) | 0.84 | 0.81 |
| Anxiety | 107 (49%) | 258 (40%) | 0.07 | 0.23 |
| Changes in libido | 60 (28%) | 230 (35%) | 0.12 | 0.45 |
| Difficulty having sex | 47 (22%) | 199 (31%) | 0.07 | 0.62 |
| Lightheadedness/dizziness | 119 (55%) | 299 (46%) | 0.10 | 0.211 |
| Falling | 30 (14%) | 113 (17%) | 0.39 | 0.425 |
| Daytime sleepiness | 48 (22%) | 123 (19%) | 0.47 | 0.33 |
| Insomnia | 152 (70%) | 413 (64%) | 0.22 | 0.46 |
| Vivid or intense dreams | 75 (35%) | 214 (33%) | 0.79 | 0.81 |
| Dream enactment | 64 (29%) | 179 (28%) | 0.76 | 0.61 |
| Leg restlessness | 114 (53%) | 312 (48%) | 0.39 | 0.43 |
| Leg swelling | 41 (19%) | 98 (15%) | 0.36 | 0.81 |
| Excessive sweating | 69 (32%) | 143 (22%) | 0.036 | 0.23 |
| Double Vision | 47 (22%) | 100 (15%) | 0.12 | 0.18 |
| Delusions | 6 (3%) | 15 (2%) | 0.79 | 0.55 |

*NMS-Quest is missing for 105 subjects. For the 867 subjects with NMS-Quest data, the distributions for age and YSD are similar to those distributions in the entire cohort.

**Adjusted for sex and MDS-UPDRS II score

**Supplementary table 3**: Baseline characteristics among the group reporting inner tremor stratified by report of anxiety as a bothersome symptom on the PD-PROP. Values shown are N (%).

|  | ANXIETY NOT REPORTED  (N = 157) | ANXIETY REPORTED  (N = 86) | P-VALUE |
| --- | --- | --- | --- |
| Age:  <50  50 – 59  60 – 69  70 – 79  >= 80 | 11 (7%)  35 (22%)  52 (33%)  52 (33%)  7 (4%) | 5 (6%)  19 (22%)  24 (28%)  36 (42%)  2 (2%) | 0.59 |
| Years Since Diagnosis:  < 3  3 – 5  6 – 7  8 – 9  >= 10 | 76 (48%)  43 (27%)  17 (11%)  8 (5%)  13 (8%) | 40 (47%)  24 (28%)  12 (14%)  5 (6%)  5 (6%) | 0.97 |
| Sex:  Women  Men | 121 (77%)  36 (23%) | 60 (70%)  26 (30%) | 0.21 |
| Education:  High School  Associates/College  Post Graduate | 17 (11%)  90 (59%)  45 (30%) | 8 (10%)  42 (50%)  34 (40%) | 0.14 |
| MDS-UPDRS II (Quartiles):  Q1: 0 – 6  Q2: 7 – 11  Q3: 12 – 17  Q4: >=18 | 62 (44%)  39 (27%)  21 (15%)  20 (14%) | 25 (32%)  21 (27%)  21 (27%)  10 (13%) | 0.15 |
| GDS-15 score >5  No  Yes | 40 (36%)  70 (64%) | 19 (37%)  33 (63%) | 0.98 |

**Supplementary table 4**: Baseline characteristics among the group reporting inner tremor stratified by sex. Values shown are N (%).

|  | WOMEN  (N = 181) | MEN  (N = 62) | P-VALUE |
| --- | --- | --- | --- |
| Age:  <50  50 – 59  60 – 69  70 – 79  >= 80 | 10 (6%)  39 (22%)  50 (28%)  75 (41%)  7 (4%) | 6 (10%)  15 (24%)  26 (42%)  13 (21%)  2 (3%) | 0.028 |
| Years Since Diagnosis:  < 3  3 – 5  6 – 7  8 – 9  >= 10 | 89 (49%)  48 (27%)  22 (12%)  9 (5%)  13 (7%) | 27 (44%)  19 (31%)  7 (11%)  4 (6%)  5 (8%) | 0.55 |
| Education:  High School  Associates/College  Post Graduate | 20 (11%)  97 (55%)  58 (33%) | 5 (8%)  35 (57%)  21 (34%) | 0.63 |
| MDS-UPDRS II (Quartiles):  Q1: 0 – 6  Q2: 7 – 11  Q3: 12 – 17  Q4: >=18 | 68 (42%)  46 (28%)  27 (17%)  21 (13%) | 19 (33%)  14 (25%)  15 (26%)  9 (16%) | 0.15 |
| GDS-15 score >5  No  Yes | 39 (33%)  81 (68%) | 20 (48%)  22 (52%) | 0.08 |
